# Epidemiology of dengue in SAARC territory: a systematic review and meta-analysis

**DOI:** 10.1186/s13071-022-05409-1

**Published:** 2022-10-24

**Authors:** Dhan Bahadur Shrestha, Pravash Budhathoki, Bipana Gurung, Subash Subedi, Shishir Aryal, Anisha Basukala, Barun Aryal, Anurag Adhikari, Ayusha Poudel, Gopal Kumar Yadav, Mtanis Khoury, Binod Rayamajhee, Lok Bahadur Shrestha

**Affiliations:** 1 Department of Internal Medicine, Mount Sinai Hospital, Chicago, IL USA; 2 Department of Internal Medicine, Bronxcare Health System, Bronx, NY USA; 3 Chitwan Medical College, Chitwan, Nepal; 4 Nepalese Army Institute of Health Sciences, Kathmandu, Nepal; 5 Department of Emergency Medicine, Patan Academy of Health Sciences, Lalitpur, Nepal; 6 Department of Emergency Medicine, Nepal National Hospital, Kathmandu, Nepal; 7 Department of Emergency Medicine, Alka Hospital, Kathmandu, Nepal; 8 Department of Emergency Medicine, Kalaiya Hospital, Bara, Nepal; 9 School of Optometry & Vision Science, Faculty of Medicine and Health, University of New South Wales, Sydney, Australia; 10 Department of Infection and Immunology, Kathmandu Research Institute for Biological Sciences (KRIBS), Lalitpur, Nepal; 11 Department of Microbiology & Infectious Diseases, B. P. Koirala Institute of Health Sciences, Dharan, 56700 Nepal; 12 School of Medical Sciences and the Kirby Institute, University of New South Wales, Sydney, Australia

**Keywords:** Dengue, SAARC region, Dengue fever, Dengue shock syndrome

## Abstract

**Background:**

Dengue is one of the common arboviral infections and is a public health problem in South East Asia. The aim of this systematic review and meta-analysis was to evaluate the prevalence and distribution of dengue in SAARC (South Asian Association for Regional Cooperation) countries.

**Methods:**

The PubMed, PubMed Central, Embase and Scopus databases were searched for relevant studies. Statistical analysis on data extracted from the selected studied was conducted using the Comprehensive Meta-Analysis Software (CMA) version 3 software package. Proportions were used to estimate the outcome with a 95% confidence interval (CI).

**Results:**

Across all studies, among cases of suspected dengue, 30.7% were confirmed dengue cases (proportion: 0.307, 95% CI: 0.277–0.339). The seroprevalence of dengue immunoglobulin (Ig)G, IgM or both (IgM and IgG) antibodies and dengue NS1 antigen was 34.6, 34.2, 29.0 and 24.1%, respectively. Among the different strains of dengue, dengue virus (DENV) strains DENV-1, DENV-2, DENV-3 and DENV-4 accounted for 21.8, 41.2, 14.7 and 6.3% of cases, respectively. The prevalence of dengue fever, dengue hemorrhagic fever and dengue shock syndrome was 80.5, 18.2 and 1.5%, respectively. Fever was a commonly reported symptom, and thrombocytopenia was present in 44.7% of cases. Mortality was reported in 1.9% of dengue cases.

**Conclusions:**

Dengue is a common health problem in South East Asia with high seroprevalence. DENV-2 was found to be the most common strain causing infection, and most dengue cases were dengue fever. In addition, thrombocytopenia was reported in almost half of the dengue cases.

**Graphical Abstract:**

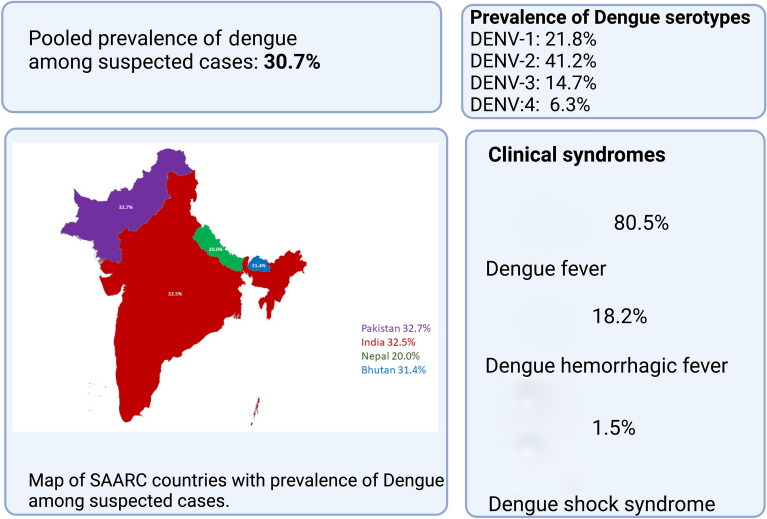

**Supplementary Information:**

The online version contains supplementary material available at 10.1186/s13071-022-05409-1.

## Background

Dengue, one of the most common arboviral infections, is transmitted by the bite of the* Aedes* mosquito. Dengue infections are caused by four circulating dengue virus serotypes (DENV-1 to -4) that are ubiquitously prevalent throughout the tropical and subtropical regions of the world. The risk of infection is strongly influenced by rainfall, temperature and the degree of urbanization [1, 2]. Dengue infection is usually asymptomatic in > 50% of cases; alternatively, it can present as a flu-like illness, including headache, myalgia and rash [3]. Therefore, knowledge of dengue's geographical distribution and burden is crucial [2]. To date, there are not licensed vaccines or specific therapeutics against dengue [2].

Dengue is one of the major public health concerns in developing countries. More than 100 countries in South East Asia, the Western Pacific region, the Americas and Africa are reported to be dengue endemic, a scenario that differs from that which prevailed 20–30 years ago [3]. Dengue is considered to be among the most significant infectious diseases, having a high disease burden, especially in the South East Asia region, with cycles of epidemics every 3–5 years [4]. Bhatt et al. estimated 390 million dengue infections worldwide in 2010, much higher than the number of cases previously assumed by WHO [2]. Shepherd et al. estimated an average of 2.9 million dengue infection episodes and 5906 deaths per year with an annual economic burden of US$950 million in the Southeast Asian region [5].

Similarly, the disability-adjusted life years (DALYS) of dengue in the South East Asian region was 372 DALYs per million per year [5]. This trend is projected to rise further in the future owing to the rapid population growth in the region together with unplanned urbanization and industrialization, increased population sensitivity and lack of licensed vaccines and specific therapeutics against dengue [2, 4]. Although all eight countries in the South Asian Association for Regional Cooperation (SAARC) region reported sporadic cases or outbreaks of dengue infection with significant health and economic burden, no has yet study scrutinized its prevalence and risk factors among the febrile and healthy general population in the SAARC region to date. The aim of our meta-analysis was, therefore, to evaluate the prevalence, risk factors and distribution of dengue fever in SAARC countries.

## Methods

### Protocol registration

The systematic review was registered in PROSPERO (CRD42020215737) and was conducted according to the Meta-Analysis of Observational Studies in Epidemiology (MOOSE) guidelines [6, 7]. For details, see Additional file 1: Text 1.

### Data sources and search strategy

The PubMed, PubMed Central, Scopus and Embase databases were searched for relevant articles from 1995 up to December 2020 using the appropriate terms and Boolean operators. For details, see Additional file 2: Text 1.

### Eligibility and exclusion criteria

The eligibility criteria for inclusion were: (i) all papers (cross-sectional studies, case series reporting > 50 patients with dengue, cohort study) mentioning prevalence of dengue and/or details of dengue-like risk factors, outcome and outcome predictors; (ii) studies conducted between 1995 onwards to date; (iii) published articles. The following studies were excluded: (i) editorials, comments, and viewpoint articles with no proper data on dengue and lacking adequate data of interest; and (ii) studies conducted outside SAARC countries. When ≥ 2 studies were identified that used the same dataset, we considered the most comprehensive and updated one for inclusion.

### Study selection

The studies were filtered using Covidence [8]. Three reviewers (BG, SS, AB) independently screened the title and abstract based on the inclusion criteria. Discrepancies were resolved by consensus, with a fourth reviewer (SA) making the decision when consensus could not be reached.

### Risk of bias assessment based on the critical appraisal checklist

Qualitative assessment of each individual study was conducted using the Joanna Briggs Institute (JBI) critical appraisal tool [9]. This checklist consists of nine items that assess the methodological quality of an investigation and determine the extent to which a study has addressed the possibility of bias in its design, conduct and analysis. Our bias assessment of the 55 articles included in our meta-analysis is shown in Table 1.

**Table 1:** Bias assessment of the studies included in the meta-analysis

Country	Rreference	JBI critical appraisal checklist^a^								
		Was the sample frame appropriate to address the target population?	Were study participants sampled appropriately?	Was the sample size adequate?	Were the study subjects and the setting described in detail?	Was the data analysis conducted with sufficient coverage of the identified sample?	Were valid methods used for the identification of the condition?	Was the condition measured in a standard, reliable way for all participants?	Was there appropriate statistical analysis?	Was the response rate adequate, and if not, was the low response rate managed appropriately?
India	[10]	Yes	Yes	Yes	Yes	Yes	Yes	Yes	Yes	Yes
India	[11]	Yes	Yes	Yes	Yes	Yes	Yes	Yes	Yes	Yes
India	[12]	Yes	Yes	Yes	Yes	Yes	Yes	Yes	Yes	Yes
India	[13]	Yes	Yes	Yes	Yes	Yes	Yes	Yes	Yes	Yes
Pakistan	[14]	Unclear	Yes	Yes	No	Yes	Yes	Yes	Yes	Yes
India	[15]	Yes	Yes	Yes	Yes	Yes	Yes	Yes	Yes	Yes
India	[16]	Unclear	Yes	Yes	No	Yes	Yes	Yes	Yes	Yes
India	[17]	Yes	Yes	Yes	Yes	Yes	Yes	Yes	Yes	Yes
India	[18]	Yes	Yes	Yes	Yes	Yes	Yes	Yes	Yes	Yes
Sri Lanka	[19]	Yes	Yes	Yes	Yes	Yes	Yes	Yes	Yes	Yes
Pakistan	[20]	Yes	Yes	Yes	Yes	Yes	Yes	Yes	Yes	Yes
India	[21]	Yes	Yes	Yes	No	Yes	Yes	Yes	Yes	Yes
India	[22]	Yes	Yes	Yes	Yes	Yes	Yes	Yes	Yes	Yes
India	[23]	Yes	Yes	Yes	Yes	Yes	Yes	Yes	Yes	Yes
India	[24]	Yes	Yes	Yes	Yes	Yes	Yes	Yes	Yes	Yes
Pakistan	[25]	Yes	Yes	Yes	No	Yes	Yes	Yes	Yes	Yes
India	[26]	Yes	Yes	Yes	No	Yes	Yes	Yes	Yes	Yes
India	[27]	Yes	Yes	Yes	No	Yes	Yes	Yes	Yes	Yes
India	[28]	Yes	Yes	Yes	Yes	Yes	Yes	Yes	Yes	Yes
Nepal	[29]	Yes	Yes	Yes	No	Yes	Yes	Yes	Yes	Yes
India	[30]	Yes	Yes	Yes	No	Yes	Yes	Yes	Yes	Yes
Pakistan	[31]	Yes	Yes	Yes	Yes	Yes	Yes	Yes	Yes	Yes
Nepal	[32]	Yes	Yes	Yes	Yes	Yes	Yes	Yes	Yes	Yes
India	[33]	Yes	Yes	Yes	No	Yes	Yes	Yes	Yes	Yes
India	[34]	Yes	Yes	Yes	No	Yes	Yes	Yes	Yes	Yes
India	[35]	Yes	Yes	Yes	Yes	Yes	Yes	Yes	Yes	Yes
India	[36]	Yes	Yes	Yes	Yes	Yes	Yes	Yes	Yes	Yes
Pakistan	[37]	Yes	Yes	Yes	No	Yes	Yes	Yes	Yes	Yes
Sri Lanka	[38]	Yes	Yes	Yes	Yes	Yes	Yes	Yes	Yes	Yes
Nepal	[39]	Yes	Yes	Yes	No	Yes	Yes	Yes	Yes	Yes
India	[40]	Yes	Yes	Yes	Yes	Yes	Yes	Yes	Yes	Yes
India	[41]	Yes	Yes	Yes	Yes	Yes	Yes	Yes	Yes	Yes
Pakistan	[42]	Yes	Yes	Yes	Yes	Yes	Yes	Yes	Yes	Yes
Pakistan	[43]	Yes	Yes	Yes	Yes	Yes	Yes	Yes	Yes	Yes
Bangladesh	[44]	Yes	Yes	Yes	No	Yes	Yes	Yes	Yes	Yes
India	[45]	Yes	Yes	Yes	No	Yes	Yes	Yes	Yes	Yes
India	[46]	Yes	Yes	Yes	Yes	Yes	Yes	Yes	Yes	Yes
Nepal	[47]	Yes	Yes	Yes	Yes	Yes	Yes	Yes	Yes	Yes
India	[48]	Yes	Yes	Yes	No	Yes	Yes	Yes	Yes	Yes
India	[49]	Yes	Yes	Yes	Yes	Yes	Yes	Yes	Yes	Yes
India	[50]	Yes	Yes	Yes	Yes	Yes	Yes	Yes	Yes	Yes
Nepal	[51]	Yes	Yes	Yes	Yes	Yes	Yes	Yes	Yes	Yes
Sri Lanka	[52]	Yes	Yes	Yes	Yes	Yes	Yes	Yes	Yes	Yes
India	[53]	Yes	Yes	Yes	Yes	Yes	Yes	Yes	Yes	Yes
Bangladesh	[54]	Yes	Yes	Yes	Unclear	Yes	Yes	Yes	Yes	Yes
India	[55]	Yes	Yes	Yes	Yes	Yes	Yes	Yes	Yes	Yes
India	[56]	Yes	Yes	Yes	Yes	Yes	Yes	Yes	Yes	Yes
Nepal	[57]	Yes	Yes	Yes	Yes	Yes	Yes	Yes	Yes	Yes
Sri Lanka	[58]	Yes	Yes	Yes	Yes	Yes	Yes	Yes	Yes	Yes
Pakistan	[59]	Yes	Yes	Yes	Yes	Yes	Yes	Yes	Yes	Yes
India	[60]	Yes	Yes	Yes	Yes	Yes	Yes	Yes	Yes	Yes
India	[61]	Yes	Yes	Unclear	Yes	Yes	Yes	Yes	Yes	Yes
India	[62]	Yes	Yes	Yes	Yes	Yes	Yes	Yes	Yes	Yes
India	[63]	Yes	Yes	Yes	Yes	Yes	Yes	Yes	Yes	Yes
Bhutan	[64]	Yes	Yes	Yes	Yes	Yes	Yes	Yes	Yes	Yes

^a^The quality of each study was assessed using the Joanna Briggs Institute (JBI) critical appraisal tool [9] that consists of the 9 items listed in the header

### Subgroup analysis

Subgroup analyses were conducted based on countries.

## Results

**Fig. 1:**
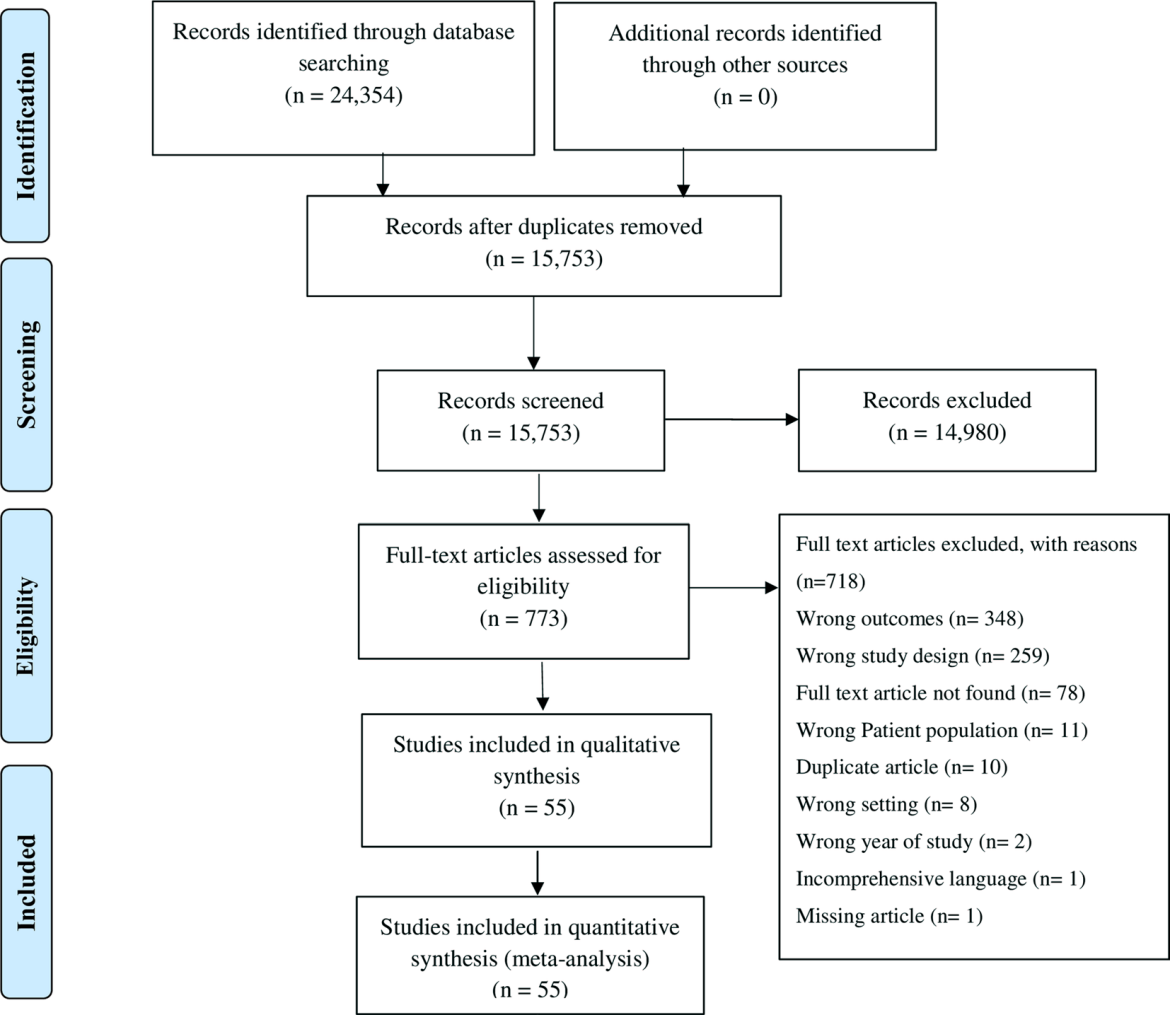
PRISMA (Preferred Reporting Items for Systematic Reviews and Meta-Analyses) flow diagram of study selection process

Our thorough search of the databases resulted in the identification of 24,354 studies. Following removal of all duplicate studies, we screened the, titles and abstracts of 15,773 studies, resulting in the exclusion of 14,980 studies; the eligibility of the remaining 773 studies was assessed on the basis of the full text. Ultimately, 55 studies were included in the final quantitative analysis (Fig. 1). The narrative summary is presented in Table 2.

**Table 2:** Narrative summary of included studies

Country	Reference	Study design	Sample size total (*n*)	Clinically suspected cases (*n*)	Laboratory-confirmed cases (*n*)	Age, years^a^	Distribution of patients by sex		Dengue infection according to DENV serotypes (*n*)			
							Male (*n*)	Female (*n*)	DENV1	DENV2	DENV3	DENV4
India	[10]	Prospective, cross-sectional	398	398	150	35.5 ± 11.6	201/398	197/398	6/60	25/60	7/60	22/60
India	[11]	Retrospective, cross-sectional	1593	1593	686	19 ± 17	968/1593	625/1593	NA	NA	NA	NA
India	[12]	Retrospective case–control	50	NA	50	NA	29/50	21//50	NA	NA	NA	NA
India	[13]	Cross-sectional	712	712	433	NA	370/712	342/712	191/433	115/433	89/433	38/433
Pakistan	[14]	Retrospective, cross-sectional	482	482	172	25.9 ± 12.8 (confirmed cases only)	96/172	76/172	NA	NA	NA	NA
India	[15]	Retrospective, cross-sectional	3677	3677	503	NA	NA	NA	NA	NA	NA	NA
India	[16]	Cross-sectional	948	NA	948	NA	671/948	277/948	NA	NA	NA	NA
India	[17]	Cross-sectional	4370	4370	1700		1046/1700	654/1700	33/55	1/55	0/55	0/55
India	[18]	Cross-sectional	8138	8138	1600		NA	NA	NA	NA	NA	NA
Sri Lanka	[19]	Cross-sectional	1167	NA	1167	32.9 ± 15	773/1167	394/1167	0/32	28/32	4/32	0/32
Pakistan	[20]	Cross-sectional	483	483	110	NA	70/110	40/110	0/17	5/17	2/17	10/17
India	[21]	Cross-sectional	4366	4366	1802	NA	2653/4366	1713/4366	NA	NA	NA	NA
India	[22]	Retrospective, cross-sectional	216	NA	216	NA	136/216	80/216	NA	NA	NA	NA
India	[23]	Cross-sectional	4019	4019	886	NA	564/886	322/886	39/103	26/103	38/103	0/103
India	[24]	Cross-sectional	1980	1980	733	NA	1335/1980	645//1980	140/733 (monotypic 63/656)	488//733 (monotypic 411/656)	185/733 (monotypic 182/656)	0/733
Pakistan	[25]	Cross-sectional	9493	9493	3504	NA	6858/9493	2635/9493	NA	NA	NA	NA
India	[26]	Retrospective, cross-sectional	309	309	34	NA	208/309	101/309	NA	NA	NA	NA
India	[27]	Cross-sectional	289	289	114	NA	84/114	30/114	NA	NA	NA	NA
India	[28]	Cross-sectional	192	192	143	NA	102/192	90/192	0/5	0/5	2/5	3/5
Nepal	[29]	Cross-sectional	283	283	28	NA	155/283	128/283	NA	NA	NA	NA
India	[30]	Prospective, observational	182	NA	182	30 ± 12.6	125/182	57/182	NA	NA	NA	NA
Pakistan	[31]	Cross-sectional	310	NA	310	32.67 ± 16.5	198/310	112/310	NA	NA	NA	NA
Nepal	[32]	Cross-sectional	266	266	45	NA	169/266	97/266	NA	NA	NA	NA
India	[33]	Cross-sectional	3312	3312	1107	NA	2054/3312	1258/3312	NA	NA	NA	NA
India	[34]	Cross-sectional	4948	4948	735	NA	502/735	233/735	3/40	6/40	13/40	1/40
India	[35]	Cross-sectional	2169	2169	412	NA	1356/2169	813/2169	NA	NA	NA	NA
India	[36]	Cross-sectional	5536	5536	1536	NA	NA	NA	0/60	47/60	1/60	2/60
Pakistan	[37]	Cross-sectional	612	612	319	NA	489/612	123/612	NA	NA	NA	NA
Sri Lanka	[38]	Cross-sectional	295	NA	295	NA	153//295	142//295	0/225	219/225	3/225	3/225
Nepal	[39]	Cross-sectional	1215	1215	403	Median 29.5 (IQR 21.3:40.0)	645/1215	570/1215	58/91	25/91	2/91	5/91
India	[40]	Cross-sectional	1668	1668	302	NA	191/302	111/302	NA	NA	NA	NA
India	[41]	Prospective, observational	313	313	137	Median 36.0 (IQR 26.0:52.0)	75/137	62/137	NA	NA	NA	NA
Pakistan	[42]	Retrospective (epidemic report)	120,948	120,948	24,938	26 ± 19.8	16,294/24938	8664/24938	NA	NA	NA	NA
Pakistan	[43]	Retrospective, descriptive	841	NA	841	31.3 ± 14.0	665/841	176/841	NA	NA	NA	NA
Bangladesh	[44]	Cross-sectional	720	NA	69	Median 12 (IQR 4:28)	454/720	268/720	NA	NA	NA	NA
India	[45]	Retrospective, cross-sectional	640	640	398	NA	380/640	260/640	NA	NA	NA	NA
India	[46]	Cross-sectional	2502	2502	464	NA	268/464	196/464	NA	NA	NA	NA
Nepal	[47]	cross sectional	239	239	70	NA	132/239	107/239	NA	NA	NA	NA
India	[48]	Retrospective, cross-sectional	55	NA	55	NA	33/55	22/55	NA	NA	NA	NA
India	[49]	Cross-sectional	536	536	112	NA	77/112	35/112	NA	NA	NA	NA
India	[50]	Retrospective, cross-sectional	219	219	135	8.3 ± 3.5	77/135	58/135	NA	NA	NA	NA
Nepal	[51]	Cross-sectional	198	198	42	45.75 ± 38.61	126/198	72/198	0/15	15/15	0/15	0/15
Sri Lanka	[52]	Prospective, observational	108	NA	108	26.6 ± 9.9	64/108	44/108	1/7	1/7	5/7	0/7
India	[53]	Retrospective, cross-sectional	5102	5102	1074	NA	664/1074	410/1074	0/3	1/3	0/3	0/3
Bangladesh	[54]	Cross-sectional	319	NA	319	33 ± 14.07	223/319	97/319	NA	NA	NA	NA
India	[55]	Retrospective analysis	900	900	461	NA	595/900	305/900	NA	NA	NA	NA
India	[56]	Cross-sectional	1426	1426	423	19 ± 17	807/1417	610/1417	NA	NA	NA	NA
Nepal	[57]	Cross-sectional	221	221	34	NA	126/221	95/221	NA	NA	NA	NA
Sri Lanka	[58]	Observational	404	NA	183	NA	108/183	75/183	NA	NA	NA	NA
Pakistan	[59]	Cross-sectional	5592	NA	5592	NA	3882/5592	1770/5592	NA	NA	NA	NA
India	[60]	Prospective, observational	81	NA	81	NA	55/81	26/81	NA	NA	NA	NA
India	[61]	Retrospective	62	NA	62	23.6 ± 3.53	0/62	62/62	NA	NA	NA	NA
India	[62]	Community-based, descriptive	2125	NA	226	NA	932/2125	1193/2125	NA	NA	NA	NA
India	[63]	Retrospective	858	NA	858	NA	NA	NA	NA	NA	NA	NA
Bhutan	[64]	Cross-sectional	379	379	119	29 ± 2	192/379	187/379	53/58	3/58	2/58	0/58

*DENV *Dengue virus,* NA* not available,* SD* standard deviation

^a^Age is presented as the mean ± standard deviation, or as the median with the interquartile range (IQR) in parentheses

### Confirmed dengue-positive cases

**Fig. 2:**
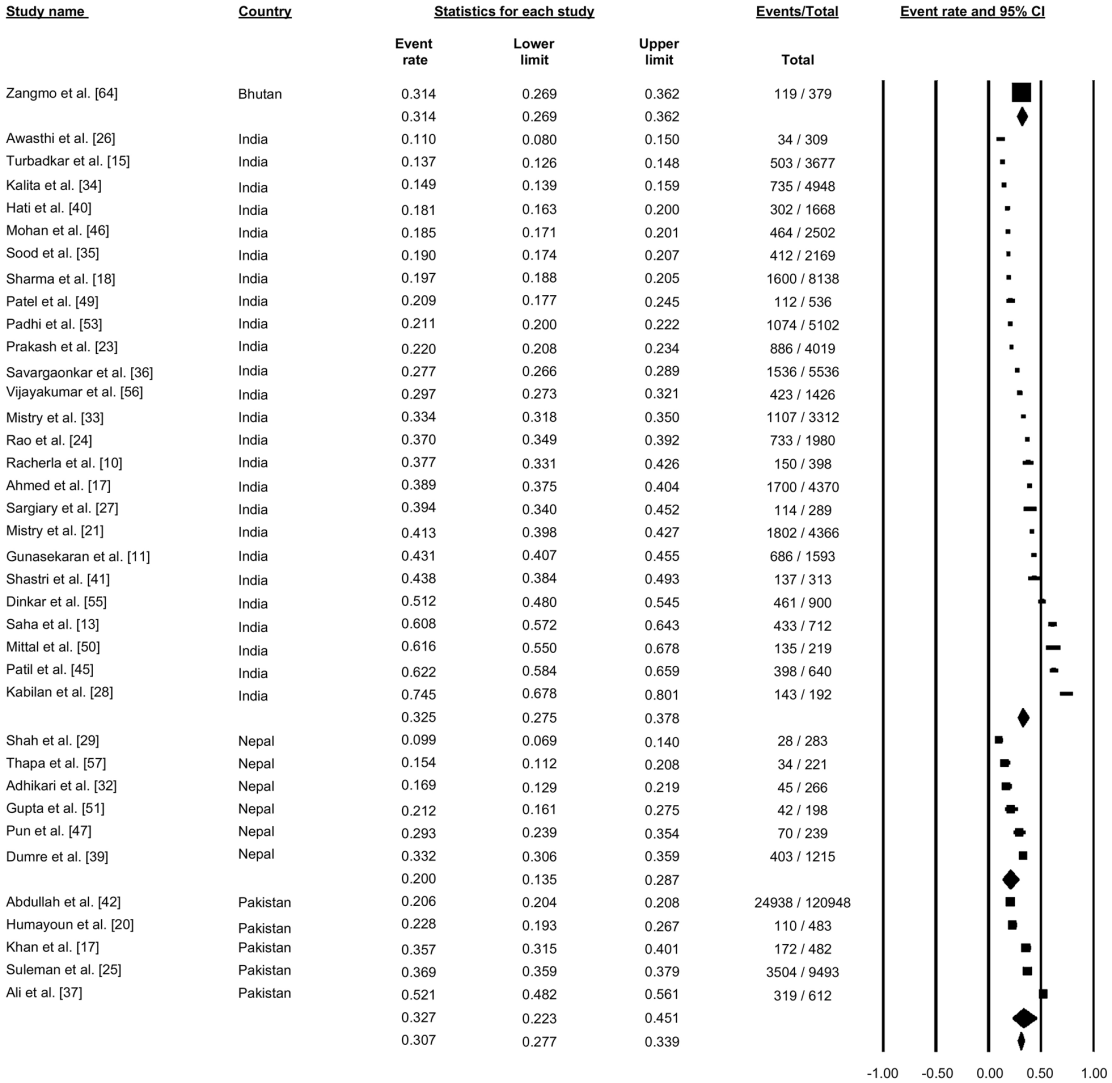
Forest plot showing the proportion of dengue cases across different countries among suspected cases of dengue using a random-effect model. Abbreviation: CI confidence, interval

A total of 37 studies reported confirmed dengue cases among suspected cases of dengue (Fig. 2). Pooling of the data using a random-effects model showed that 30.7% (proportion: 0.307, 95% CI: 0.277–0.339, *I*^2^: 99.42%) of the suspected cases were confirmed to be dengue. The proportion of confirmed cases varied from country to country in the SAARC countries. In Bhutan, one study reported that the proportion of confirmed dengue cases was 31.4%. In India, 25 studies reported confirmed dengue cases among suspected cases, with the proportion of confirmed cases ranging from 11% to 74.5% across the studies; the pooled proportion of confirmed cases across all studies was 32.5% (progression: 0.325, 95% CI: 0.275–0.378). In Nepal, six studies reported confirmed dengue cases among the suspected cases, with the proportion ranging from 9.9% to 33.2% across different studies; the pooled proportion of confirmed cases was 20% (proportion: 0.200, 95% CI: 0.135–0.287). Five studies from Pakistan reported the proportion of confirmed cases among suspected dengue cases, with a range of 20.5% to 52.1%; the pooled proportion of confirmed cases across all studied was 32.7% (proportion: 0.327, 95% CI: 0.223–0.458) (Fig. 2).

### Dengue immunoglobulin M seroprevalence

**Fig. 3:**
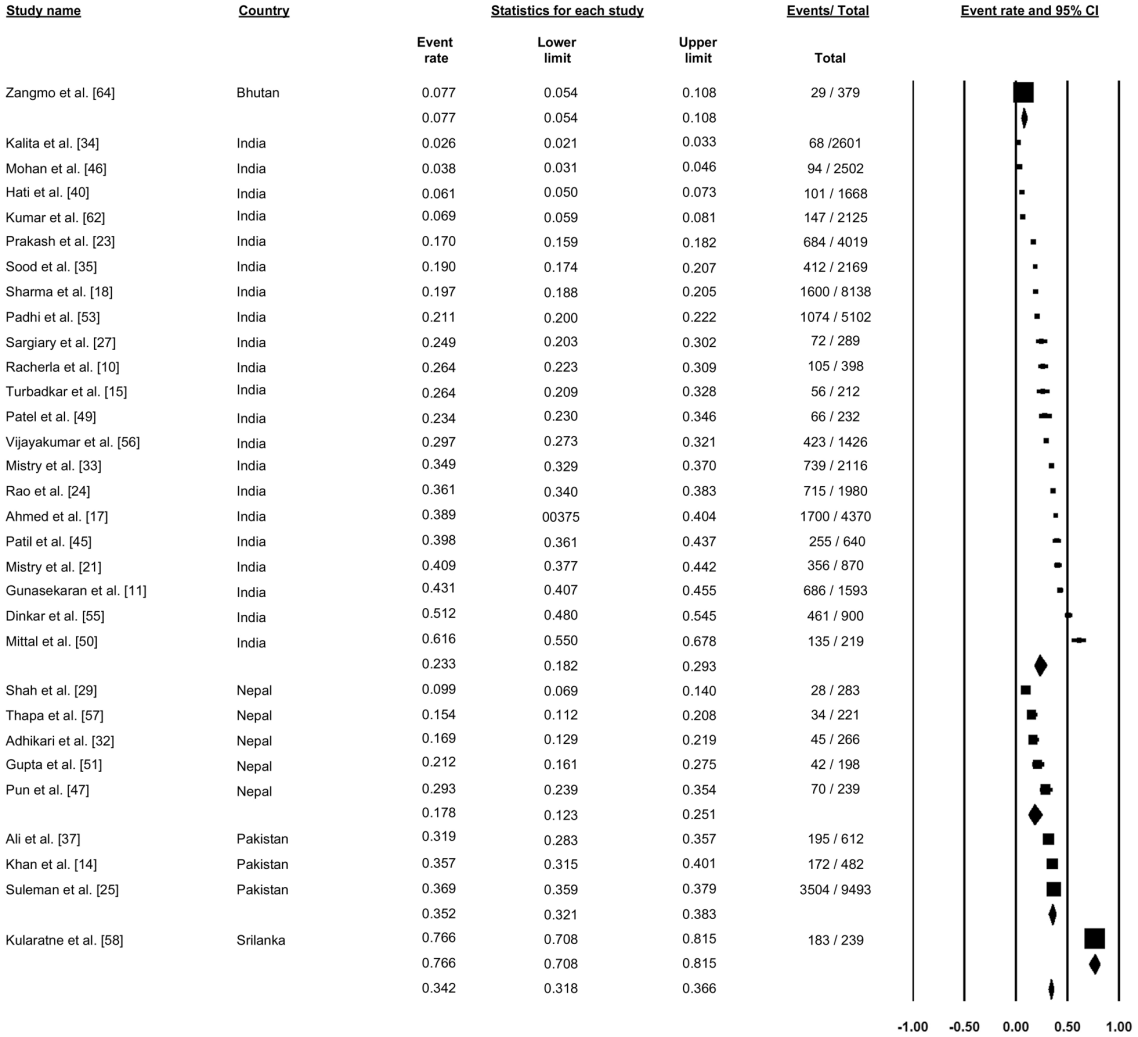
Forest plot showing the proportion of seroprevalence of dengue immunoglobulin M (IgM) antibodies across different countries among suspected cases of dengue using a random-effect model

A total of 31 studies reportedly the seroprevalence of dengue immunoglobulin M (IgM) antibodies (Fig. 3). Pooling the data using a random effect model across all the studies showed a seroprevalence of 34.2% (proportion: 0.342, 95% CI: 0.318–0.366, *I*^2^: 99.26%). One study from Bhutan showed a seroprevalence of 7.7%, and 21 studies from India reported seroprevalence ranging from 2.6% to 61.6%, with a pooled dengue IgM seroprevalence of 23.3% (proportion: 0.233, 95% CI: 0.182–0.293). Five studies reported seroprevalence from Nepal, ranging from 9.9% to 29.3%, with a pooled dengue IgM seroprevalence 17.8% (proportion: 0.178, 95% CI: 0.123–0.251). Three studies reported dengue IgM seroprevalence from Pakistan, with a pooled prevalence of 35.2% (proportion: 0.352, 95% CI: 0.321–0.383). Finally, one study from Sri Lanka showed a dengue IgM seroprevalence of 76.6% (Fig. 3).

### Dengue NS1 seroprevalence

**Fig. 4:**
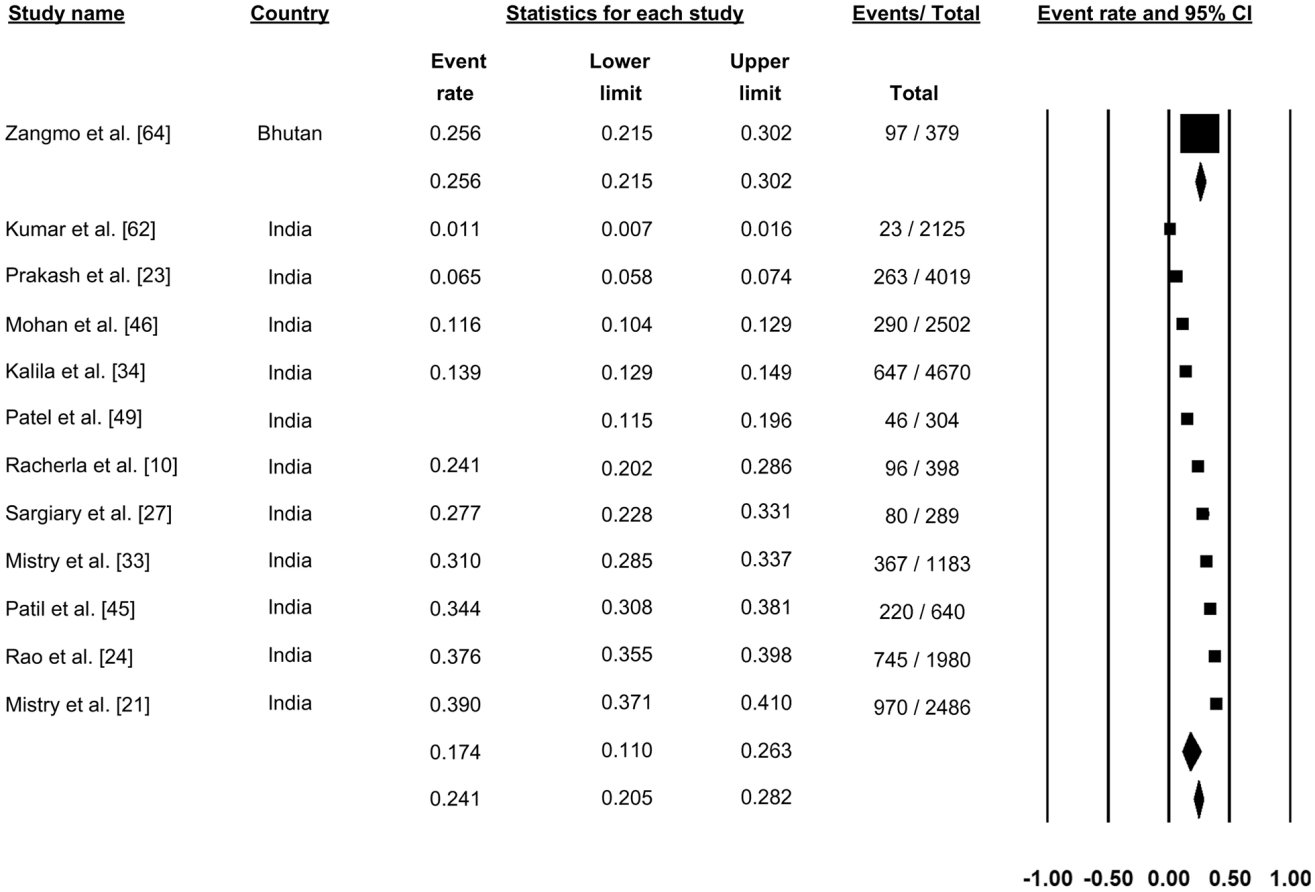
Forest plot showing the proportion of dengue IgM seroprevalence across different countries among suspected cases of dengue using a random-effect model

Twelve studies reported the seroprevalence of NS1 antigen, and pooling of these results showed dengue NS1 positivity in 24.1% of the cases (proportion: 0.241, 95% CI: 0.205–0.282, *I*^2^: 99.4) (Fig. 4).

### Dengue immunoglobulin G seroprevalence

**Fig. 5:**
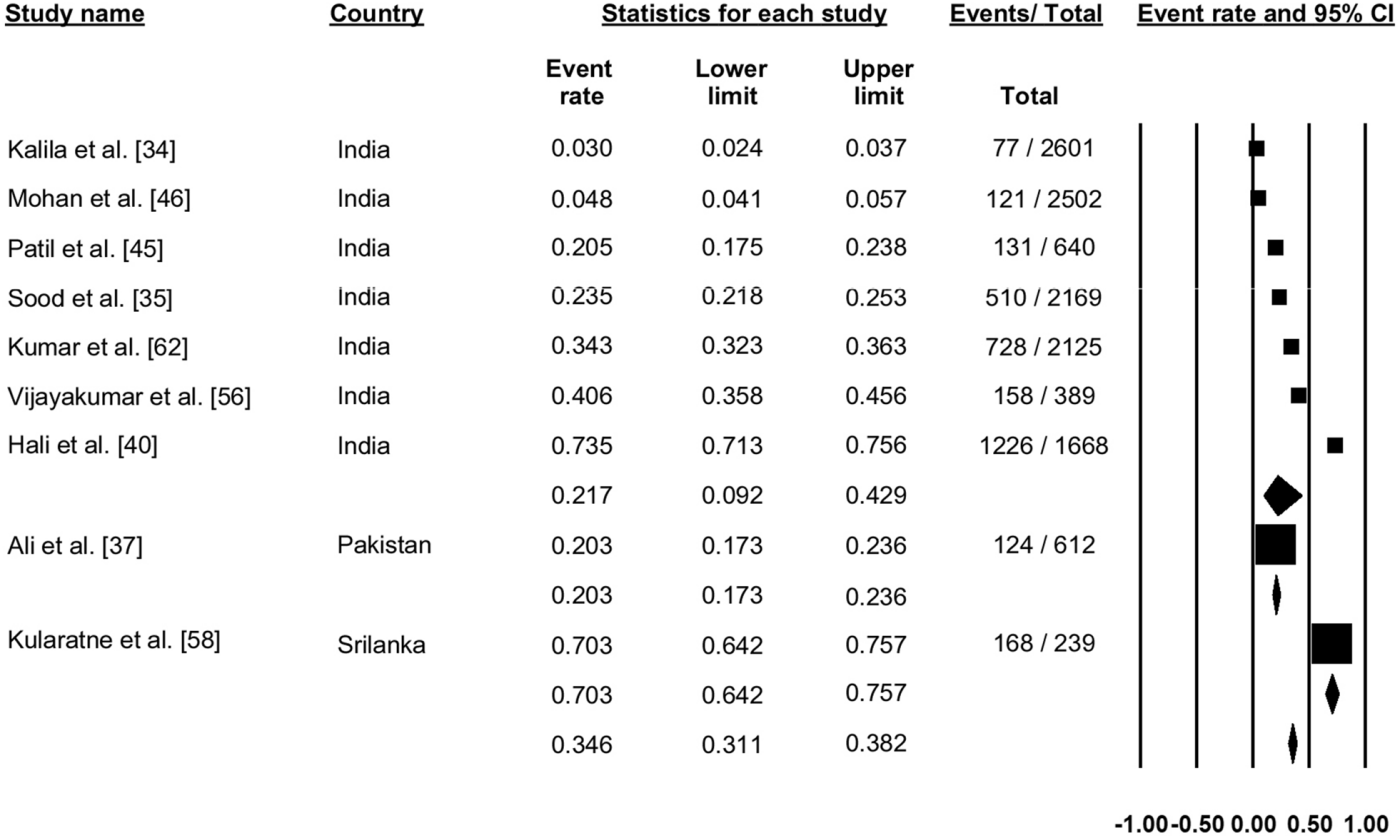
Forest plot showing the proportion of dengue immunoglobulin G (IgG) seroprevalence across different countries among suspected cases of dengue using a random-effect model

Nine studies reported the seroprevalence of dengue immunoglobulin G (IgG) antibodies (Fig. 5). Pooling of the data showed dengue IgG positivity in 34.6% of cases (proportion: 0.346, 95% CI: 0.311–0.382, *I*^2^: 99.67) (Fig. 5).

### Combined dengue IgM and IgG seroprevalence

**Fig. 6:**
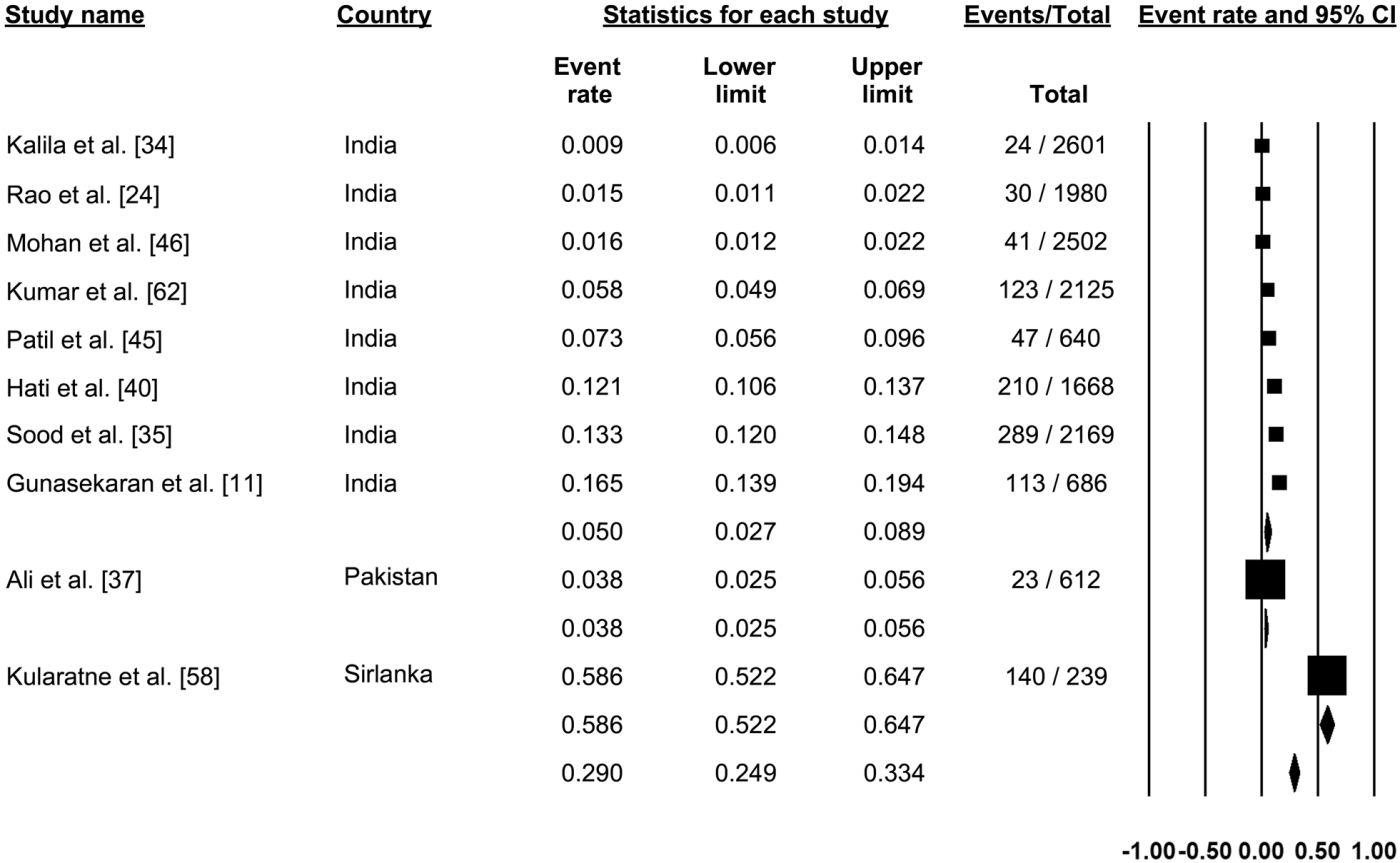
Forest plot showing the proportion of both IgM and IgG seroprevalence of dengue across different countries among suspected cases of dengue using a random-effect model

Ten studies reported seroprevalence of both IgM and IgG antibodies of dengue (Fig. 6). Pooling of these results showed positivity for both dengue IgM and IgG antibodies in 29.0% of cases (proportion: 0.290, 95% CI: 0.249–0.334, *I*^2^: 99.02) (Fig. 6).

### DENV virus strain

**Fig. 7:**
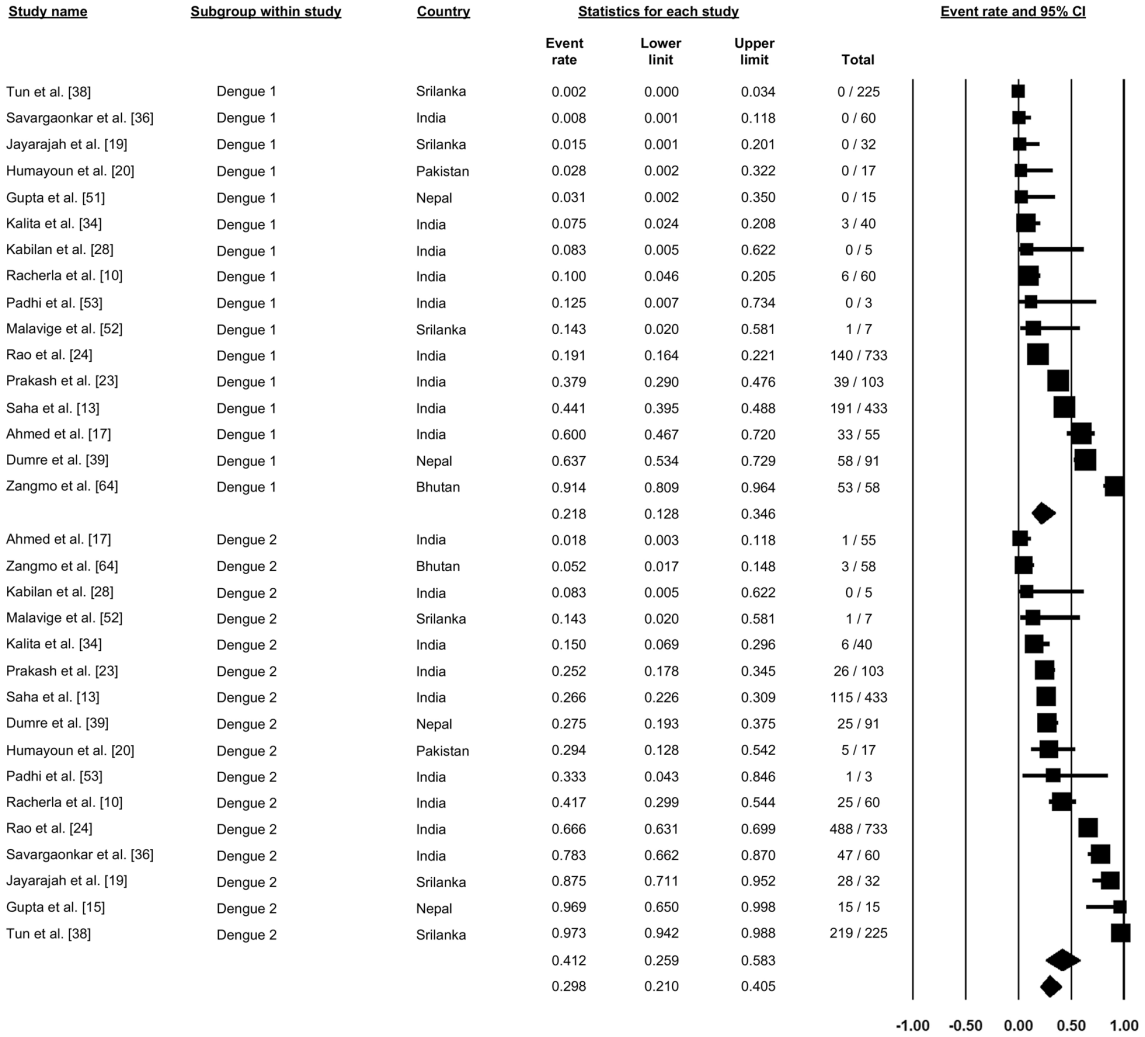
Forest plot showing the proportion of dengue virus strains 1 and 2 (DENV-1, DENV-2) across different countries among confirmed cases of dengue using a random-effect model

**Fig. 8:**
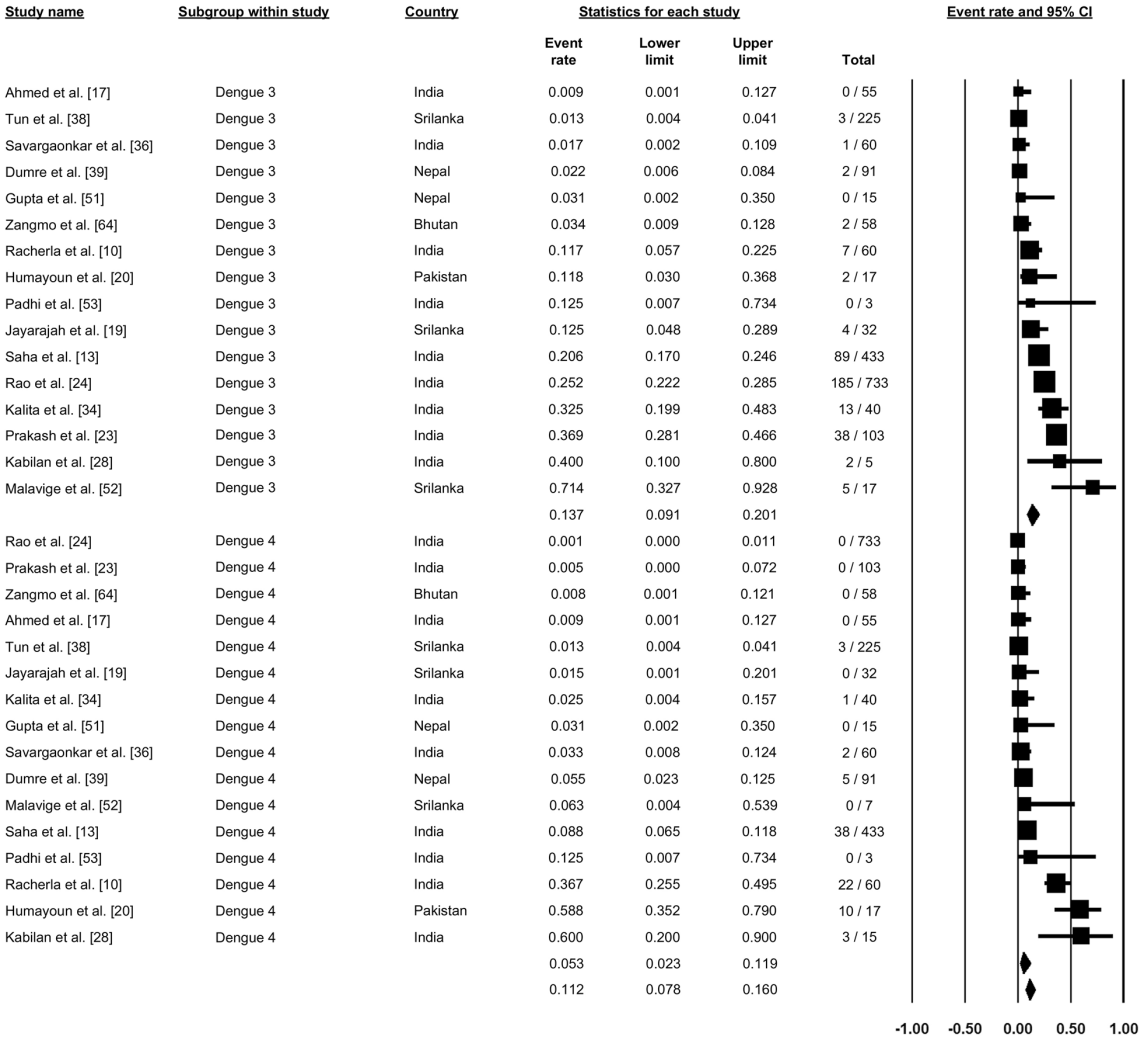
Forest plot showing the proportion of DENV-3 and DENV-4 across different countries among confirmed cases of dengue using a random-effect model

A total of 16 studies reported on the different strains of DENV, with the proportion of different strains varying between studies (Figs. 7, 8). A study from Bhutan reported that DENV-1 was the most prevalent strain in that country (91.4%). Studies from Sri Lanka, India and Nepal reported differences in the prevalence of specific strains among these three countries, with the predominant DENV strain(s) being DNV-1 and DNV-2 in Nepal, DNV-2 and DNV-3 in Sri Lanka and DNV-4 in Sri Lanka. In contrast, in India, all four strains were reported to be circulating in different parts of the country, with, overall, DENV-1 accounting for 21.8% (proportion: 0.18, 95% CI: 0.128–0.346), DENV-2 accounting for 41.2% (proportion: 0.412, 95% CI: 0.250–0.583), DENV-3 accounting for 14.7% (proportion: 0.137, 95% CI: 0.091–0.201) and DENV-4 accounting for 6.3% (proportion: 0.063, 95% CI: 0.023–0.119) (Figs. 7, 8).

### Residential setting (urban versus rural)

**Fig. 9:**
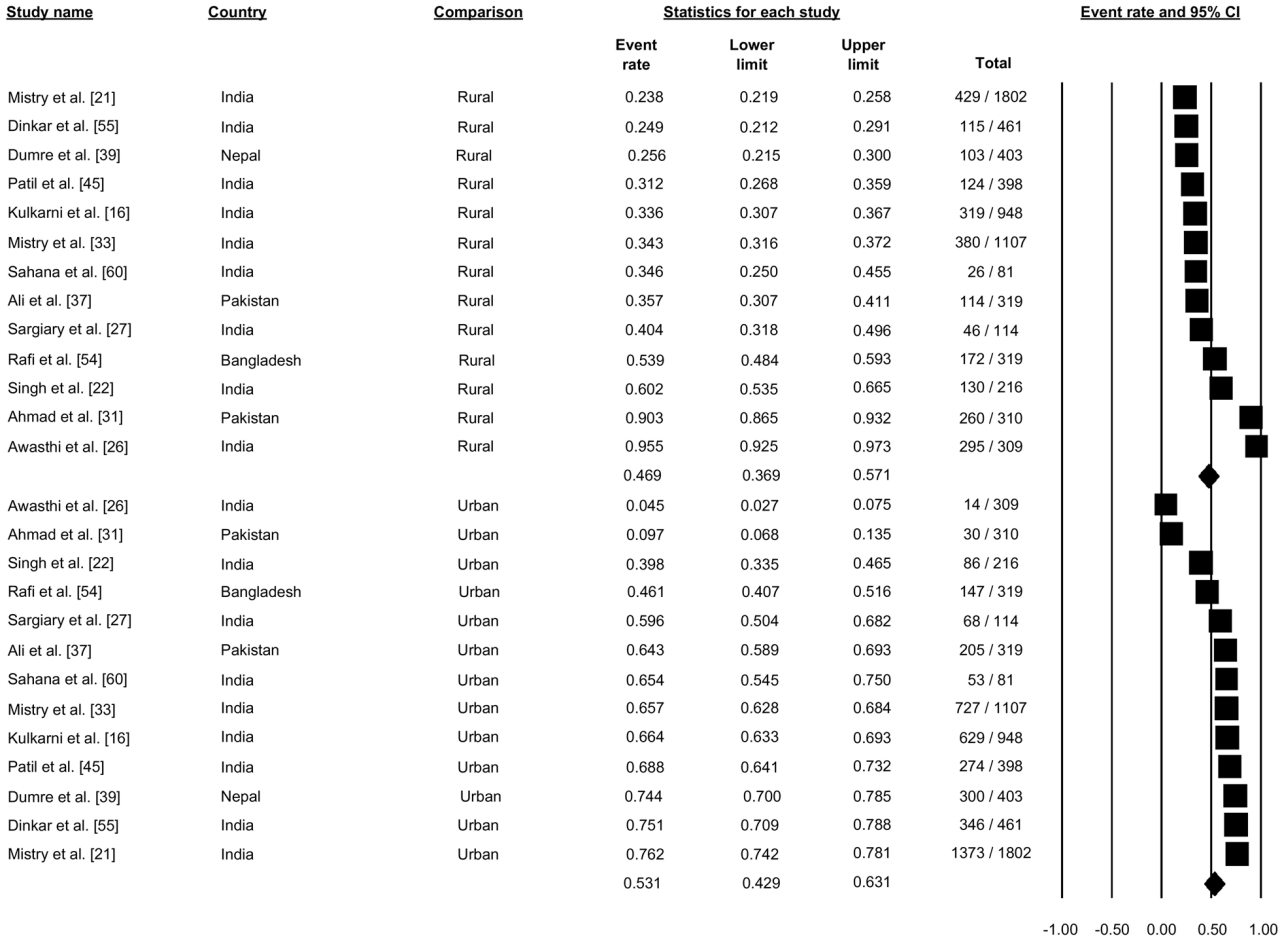
Forest plot showing the proportion of dengue cases based on residential setting across different countries among cases of dengue using a random-effect model

Thirteen studies reported the residential setting of patients with dengue (Fig. 9). Pooling of these data showed the 46.95% of dengue cases were from rural settings (proportion: 0.469, 95% CI: 0.369–0.571) and that 53.1% were from urban residential settings (proportion 0.531, 95% CI: 0.429–0.631) (Fig. 9).

### Dengue severity

#### Dengue fever

**Fig. 10:**
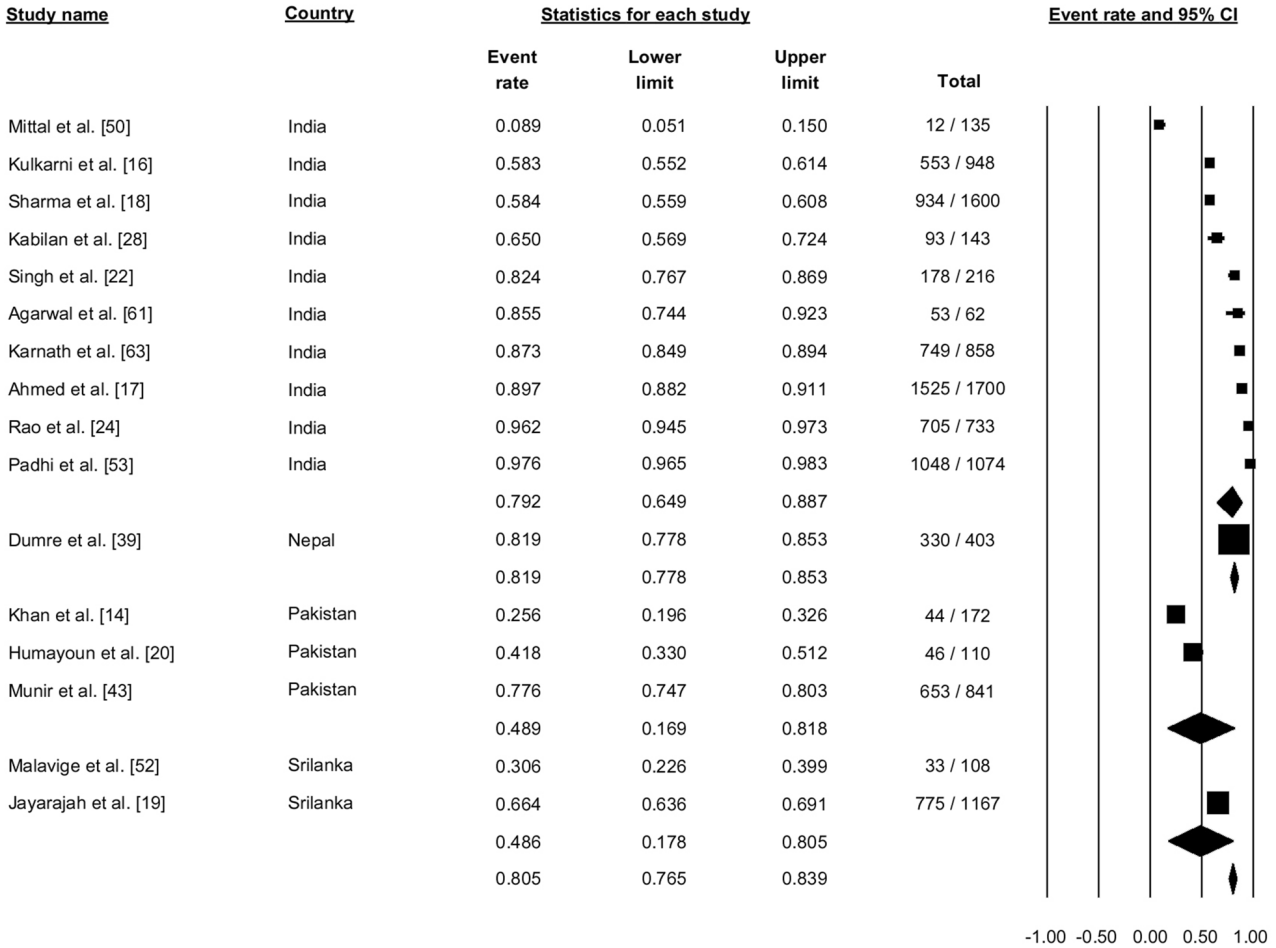
Forest plot showing the proportion of dengue fever cases across different countries among cases of dengue using a random-effects model

Sixteen studies reported dengue fever (Fig. 10). Pooling the data using the random effects model showed that 80.5% of the cases were categorized as dengue fever (proportion: 0.805, 95% CI: 0.765–0.839, *I*^2^: 98.86) (Fig. 10).

#### Dengue hemorrhagic fever

**Fig. 11:**
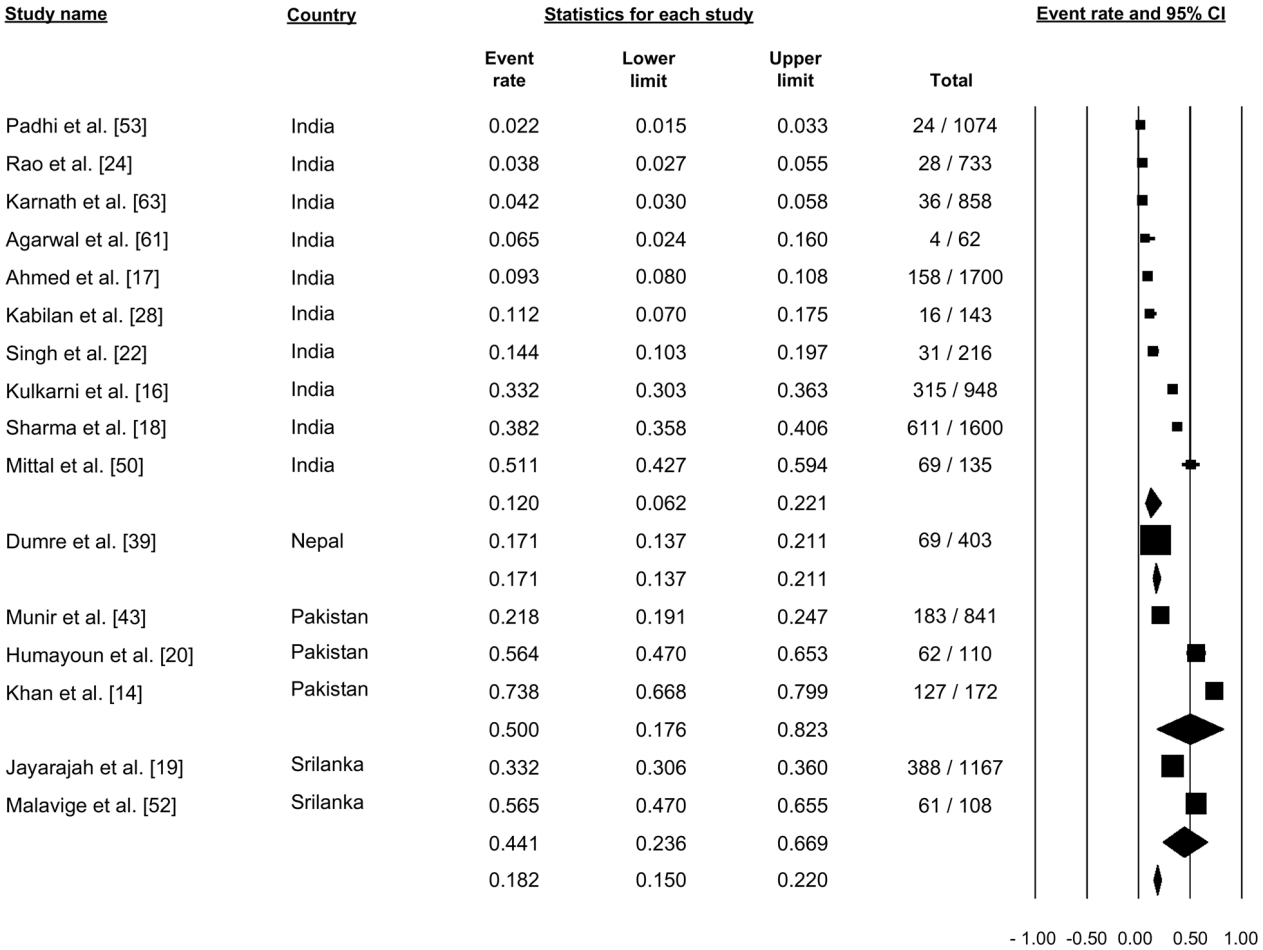
Forest plot showing the proportion of dengue hemorrhagic fever cases across different countries among cases of dengue using a random-effects model

Sixteen studies reported dengue hemorrhagic fever (DHF) (Fig. 11). Pooling of the data using the random-effects model showed that 18.2% of the cases were categorized as DHF (proportion: 0.182,, 95% CI: 0.150–0.220, *I*^2^: 98.73) (Fig. 11).

#### Dengue shock syndrome

**Fig. 12:**
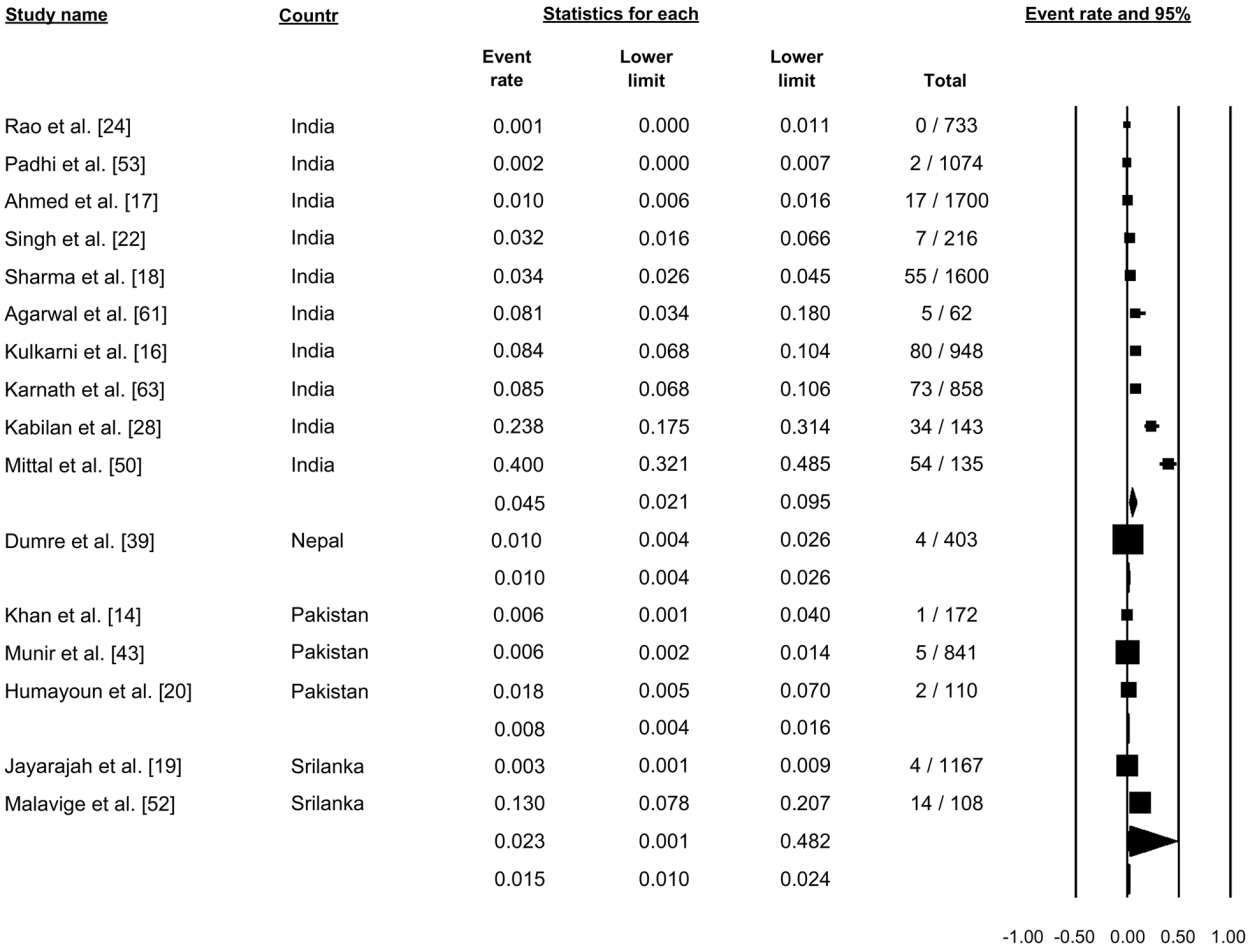
Forest plot showing the proportion of dengue shock syndrome cases across different countries among cases of dengue using a random-effects model

Sixteen studies reported dengue shock syndrome (Fig. 12). Pooling of the data using a random-effects model showed that 1.5% of the cases were categorized as dengue shock syndrome (proportion: 0.015, 95% CI: 0.010–0.024, *I*^2^: 96.70) (Fig. 12).

### Fever as a symptom

**Fig. 13:**
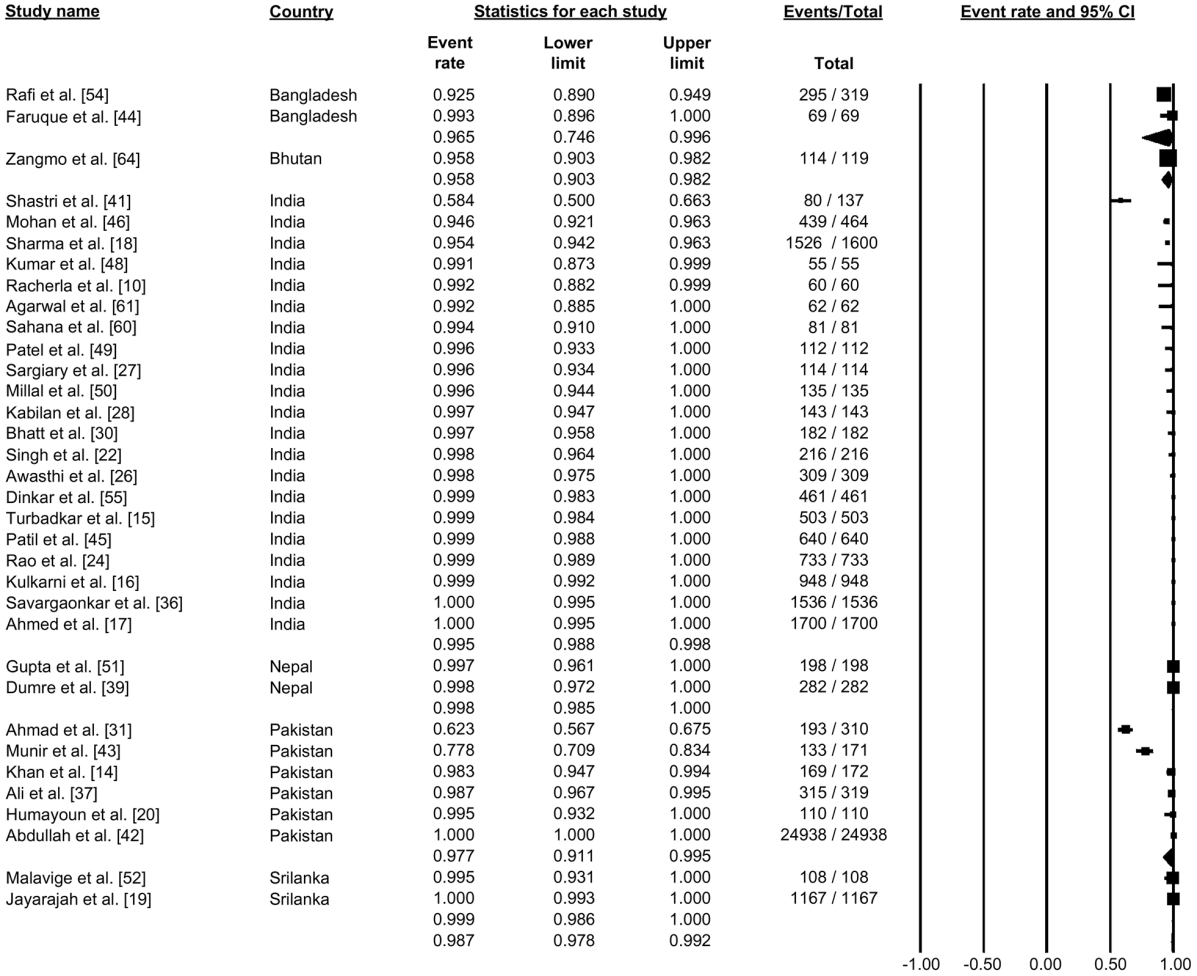
Forest plot showing the proportion of fever among cases of dengue across different countries using a random-effects model

A total of 34 studies reported the status of fever as a symptom (Fig. 13). Pooling the data using the random-effects model showed that 98.7% of the patients had a fever, which was the most typical symptom reported (proportion: 0.987, 95% CI: 0.978–0.992, *I*^2^: 95.06) (Fig. 13).

### Thrombocytopenia

**Fig. 14:**
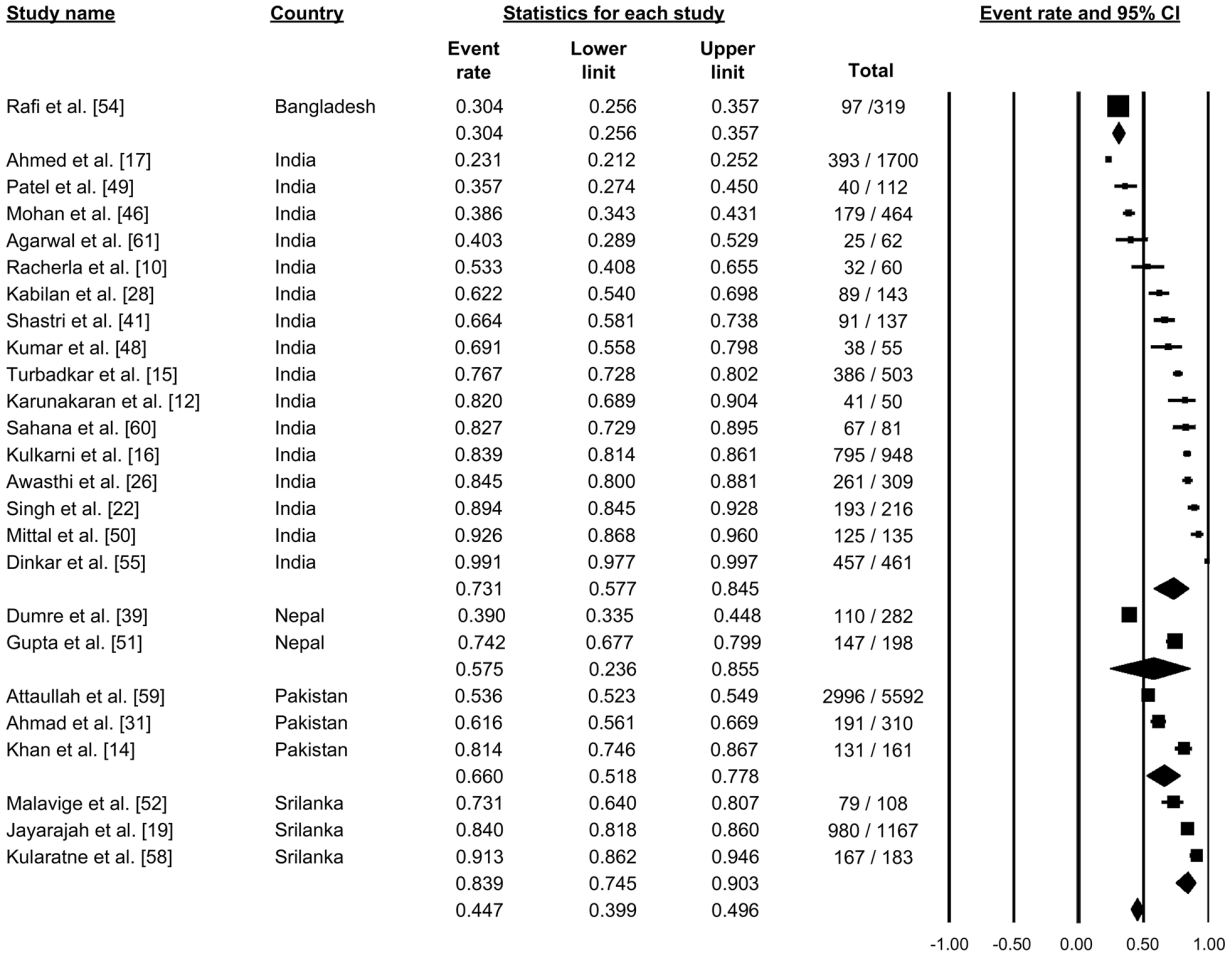
Forest plot showing the proportion of thrombocytopenia among cases of dengue across different countries using a random-effects model

A total of 25 studies reported thrombocytopenia among dengue cases (Fig. 14). Pooling these findings using the random-effects model showed that 44.7% of patients had a low platelet count (proportion: 0.447, 95% CI: 0.399–0.496, *I*^2^: 98.77) (Fig. 14).

### Mortality outcome

**Fig. 15:**
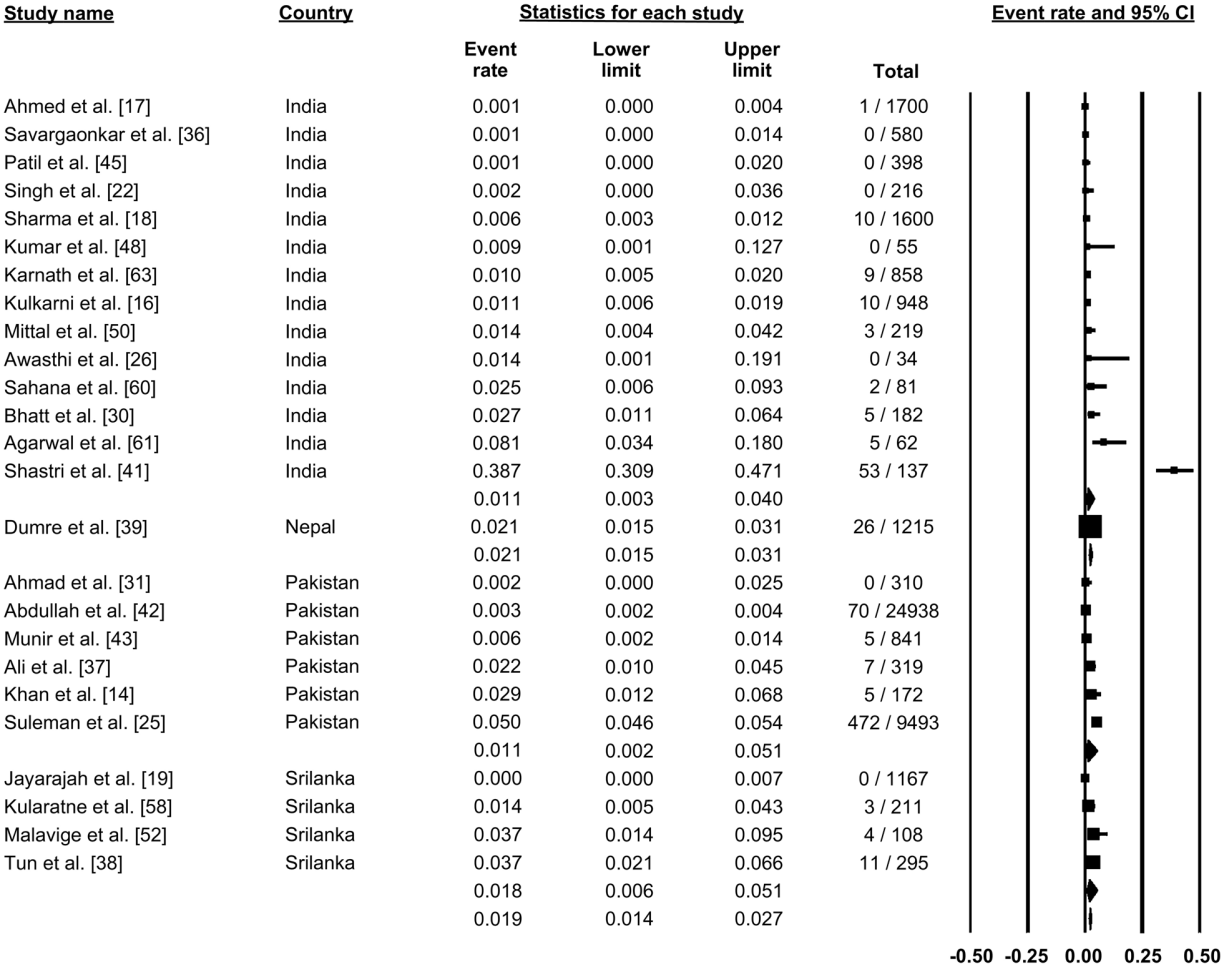
Forest plot showing mortality among cases of dengue across different countries using a random-effects model

Mortality was reported in 25 studies (Fig. 15). Pooling these data using a random-effects model showed that mortality was reported in 1.9% of dengue cases (proportion: 0.019, 95% CI: 0.014–0.027, *I*^2^: 97.4) (Fig. 15).

### Publication bias

Publication bias is among the different outcomes assessed using the Funnel plot and Egger’s test. Rough estimates for publication bias were based on the symmetry of the Funnel plot and confirmed using the Egger’s test. Publication bias was significant for the confirmed dengue-positive cases and the ‘fever as a symptom’ outcome (*P* < 0.05); for the remaining other outcomes, publication bias was not significant (*P* > 0.05).

## Discussion

The present study reports on the epidemiology of dengue infection in SAARC countries over the past 2.5 decades. There was a substantial discrepancy in the proportion of laboratory-confirmed dengue infections among the SAARC countries, with the highest proportion reported in Pakistan and the lowest reported in Nepal. The overall pooled proportion of confirmed cases among suspected cases was 30.7%, with a significantly increased heterogeneity that varies according to country and time frame of the studies, infection marker of interest, severity and outcome [65]. This prevalence is very high compared to the pooled proportion of autochthonous dengue infections in Europe (0.7%). In a systematic review study by Ganeshkumar et al. in 2018, the pooled proportion of dengue infections in India was 38.3% [66]. We were unable to determine the proportion of dengue infections in a number of SAARC countries, including Afghanistan, Bangladesh, Maldives and Sri Lanka, due to the lack of published studied from these countries.

In this study, the pooled seroprevalence of dengue infection based on markers, namely IgM and IgG antibodies and NS1 antigen was 34.2, 35.2 and 24.1%, respectively. In comparison, the pooled seroprevalence of dengue infection based on both IgM and IgG seropositivity was 29.0%. These values are similar to those reported by Li et al., who found that the worldwide seroprevalence of dengue infection was 38%, with the highest dengue seroprevalence, namely 56%, in the South East Asia region and the lowest, namely 4%, in the European arena [67]. However, IgM, IgG and DENV-RNA pooled seroprevalence in febrile participants of Africa was reported to be 8.4, 10.8 and 24.8%, respectively [68]. Similarly, the seroprevalence in the general population of the Middle East and North Africa was reported by Humphrey et al. to be 25% (range: 0–62%) from 1941 to 2015 [69].

The likely explanation for the increased clinical and serological prevalence of dengue cases in SAARC countries could be the climate, geographic distribution of mosquito vectors, urbanization and absence or failure of appropriate vector control measures [70–73]. Based on a thermodynamic model, dengue virus transmission increases at a mean temperature of < 18 °C as the diurnal temperature range increases, indicating that small fluctuations in temperature favor dengue virus development [74, 75].

Our study showed that different strains of the dengue virus were predominant in different countries of SAARC. DENV-2 was most predominant dengue virus strain (41.2%), followed by DENV-1 (21.8%). The DENV-2 strain is considered to be the most virulent and life-threatening of the four serotypes [76]. The predominance of different DENV strains in different areas could be due to selection during DENV evolution, as well as viral fitness in the human or mosquito host that allows some lineages to survive better than others [77]. Therefore, the dynamics of serotype oscillations is a complex phenomenon. In our study, DENV-2 and DENV-3 were predominant in Sri Lanka. The novel appearance of DENV-3 in 1989 in Sri Lanka is considered to be responsible for the emergence of DHF in that year. [78] Similarly, in our study, DENV-4 was predominant in Pakistan, but DENV-2, DENV-3 and DENV-4 are known to have co-circulated in Pakistan from 2008 to 2011. These three serotypes share an Indian ancestry and are likely to have been introduced first into southern Pakistan. DENV-2 and DENV-3 had undergone in situ evolution to emerge as distinct, heterogeneous virus populations during the same period [79]. In the same way, all four strains were circulating in India, when a novel clade of DENV-4 (genotype I) abruptly emerged in Pune, India, during the 2016 season [80, 81].

Efforts to combat dengue infection by vaccinating susceptible and high-risk populations have been ongoing globally for over three decades, albeit without much success. Several candidate vaccines against dengue are in the pipeline [82, 83]. Since DENV-2 is known to be widely distributed across India, Nepal and Sri Lanka based on the studies included in our meta-analysis, the live attenuated CYD TDV tetravalent vaccine (Sanofi Pasteur, Lyon, France) that successfully met its targets in the phase III trial would also not be effective for SAARC countries [84] due to the resistance shown by DENV-2 to this vaccine [85, 86].

 According to the WHO 2009 classification criteria, dengue cases are classified into dengue without warning signs, dengue with warning signs and severe dengue [87, 93]. However, due to the lack of data based on the WHO 2009 classification and evidence that has become available since the WHO 1997 classification, the severity of dengue has been classified into dengue fever, dengue hemorrhagic fever and dengue shock syndrome. This study showed that most dengue infection cases were less severe.

In our study, thrombocytopenia was present in nearly 50% of all dengue cases, primarily due to bone marrow suppression and increased peripheral destruction of platelets during the febrile and early convalescent phase of the disease [88]. This result is similar to that reported in a retrospective cohort study where the prevalence of thrombocytopenia among patients with confirmed dengue cases was 40.3% [89]. The rapid decline in platelet count indicates dengue with warning signs and helps the healthcare provider to classify the dengue infection. Thrombocytopenia is also a prognostic marker, and a platelet count < 20,000/ml blood is a good predictor of mortality in patients with severe dengue [90].

The results of our study lead us to conclude that the pooled dengue case fatality rate was 1.9% in SAARC countries [66]. This contrasts with a systematic review of dengue infection in India, where the pooled estimate of case fatality rate was 2.6% [80, 81]. The most likely reason for this difference is that in India all dengue strains responsible for multiple dengue outbreaks are circulating. The mortality in dengue cases is multifactorial, determined primarily by prior health status [91].

The WHO has set a target for dengue control by reducing the case fatality rate from 0.8% in 2020 to 0.0% in 2030 [92]. The WHO roadmap against neglected tropical diseases (NTDs) has prioritized a group of 20 NTDs, including dengue fever, that requires a global collaboration of all partners to achieve the target [92]. This dramatically emphasizes the need for an in-depth understanding of the epidemiology of dengue infection to combat it in SAARC countries.

Our systematic review has certain limitations. First, we included peer-reviewed literature from selected databases and excluded gray literature that provided additional data. Secondly, age was not uniformly reported in the included studies, and we did not estimate the prevalence of dengue cases by age. Third, we were not able to categorize the severity of dengue by the WHO 2009 classification since most of the included literature used data on the severity of dengue based on the WHO 1997 classification.

## Conclusion

Dengue is common in South East Asian countries, with infected individuals showing a high seroprevalence of dengue IgG, IgM and both (IgM and IgG) antibodies and dengue NS1 antigen, in descending order or seroprevalence. DENV-2 was the most common strain, followed by DENV-1 and DENV-3; DENV-4 was the less reported strain. Fortunately, dengue fever was the most common presentation (80.5%), followed by DHF (18.2%) and dengue shock syndrome (1.5%). As the burden of dengue in the SAARC region is significant, the various governments and stakeholders need to focus on preventive measures, including vector control and timely diagnosis and treatment of the dengue cases.

## Supplementary Information

**Additional file 1: **MOOSE guidelines.

**Additional file 2: **Electronic search details.

## Data Availability

The datasets analyzed during the current study are available in the manuscript and supplement files.
