# Supplementary material for: Epidemiology of dengue in SAARC territory: a systematic review and meta-analysis
Source: Parasit Vectors. 2022 Oct 24;15:389. doi: 10.1186/s13071-022-05409-1 (PMC9594905; doi:10.1186/s13071-022-05409-1)
Supplement: Supplementary file 2 — Additional file 2: Electronic search details. [file 13071_2022_5409_MOESM2_ESM.docx]

**Electronic search details**

**Epidemiology of Dengue in The South Asian Association for Regional Cooperation (SAARC) territory: A Systematic Review and Meta-analysis**

Search builder

Terms: Dengue, Dengue fever, Dengue Shock Syndrome, Nepal, India, Bangladesh, Bhutan, Maldives, Pakistan, Sri Lanka, Afghanistan used to search relevant papers in Pubmed, PMC (Medline), Scopus, and Embase using appropriate bullions with no language restriction.

**Time: 1995 onwards**

**Pubmed search:**

(Dengue OR (Dengue fever) OR (Dengue Shock Syndrome)) and (Nepal OR India OR Bangladesh OR Bhutan OR Maldives OR Pakistan OR (Sri Lanka) OR Afghanistan)

Total hit: 3497

Link: <https://pubmed.ncbi.nlm.nih.gov/?term=%28Dengue+OR+%28Dengue+fever%29+OR+%28Dengue+Shock+Syndrome%29%29+and+%28Nepal+OR+India+OR+Bangladesh+OR+Bhutan+OR+Maldives+OR+Pakistan+OR+%28Sri+Lanka%29+OR+Afghanistan%29&filter=years.1995-2020&sort=date&size=200>

**Pubmed Central search:**

**Total hits: 12801**

**Search:** (dengue OR (dengue fever) OR (dengue shock syndrome)) and (nepal OR india OR bangladesh OR bhutan OR maladies OR pakistan OR (sri lanka) OR afghanistan)

**Link:** <https://www.ncbi.nlm.nih.gov/pmc/?term=(dengue+OR+(dengue+fever)+OR+(dengue+shock+syndrome))+and+(nepal+OR+india+OR+bangladesh+OR+bhutan+OR+maladies+OR+pakistan+OR+(sri+lanka)+OR+afghanistan)&cmd=correctspelling>

Embase search:

**Total hits: 6056**

**Details:** (dengue OR (dengue AND fever) OR (dengue AND shock AND syndrome)) AND (nepal OR india OR bangladesh OR bhutan OR maldives OR pakistan OR (sri AND lanka) OR afghanistan) AND [1995-2020]/py

**Link:** [**https://www.embase.com/#advancedSearch/resultspage/history.16/page.1/200.items/orderby.relevance/source**](https://www.embase.com/#advancedSearch/resultspage/history.16/page.1/200.items/orderby.relevance/source)**.**

**Scopus:**

**Total hits: 2000**

**Details:** (dengue OR (dengue fever) OR (dengue shock syndrome)) and (nepal OR india OR bangladesh OR bhutan OR maladies OR pakistan OR (sri lanka) OR afghanistan)

**Link:** <https://www.scopus.com/results/results.uri?sort=plf-f&src=s&st1=(Dengue%20OR%20(Dengue%20fever)%20OR%20(Dengue%20Shock%20Syndrome))%20and%20(Nepal%20OR%20India%20OR%20Bangladesh%20OR%20Bhutan%20OR%20Maldives%20OR%20Pakistan%20OR%20(Sri%20Lanka)%20OR%20Afghanistan)&nlo=&nlr=&nls=&sid=445d479662382f4a7c337f7898de905d&sot=b&sdt=cl&cluster=scopubyr%2C%222021%22%2Ct%2C%222020%22%2Ct%2C%222019%22%2Ct%2C%222018%22%2Ct%2C%222017%22%2Ct%2C%222016%22%2Ct%2C%222015%22%2Ct%2C%222014%22%2Ct%2C%222013%22%2Ct%2C%222012%22%2Ct%2C%222011%22%2Ct%2C%222010%22%2Ct%2C%222009%22%2Ct%2C%222008%22%2Ct%2C%222007%22%2Ct%2C%222006%22%2Ct%2C%222005%22%2Ct%2C%222004%22%2Ct%2C%222003%22%2Ct%2C%222002%22%2Ct%2C%222001%22%2Ct%2C%222000%22%2Ct%2C%221999%22%2Ct%2C%221998%22%2Ct%2C%221997%22%2Ct%2C%221996%22%2Ct%2C%221995%22%2Ct&sessionSearchId=445d479662382f4a7c337f7898de905d&origin=resultslist&zone=leftSideBar&editSaveSearch=&txGid=1ca5218abb69096600a78d0dd1693bb8&fbclid=IwAR271uDF7WqGz6SIsUWTT6vt6p_d9sdCt7-e5BDdVamvbzW1vBgLxEaUKTs>
